# Short Chain *N*-Acylhomoserine Lactone Production by Clinical Multidrug Resistant *Klebsiella pneumoniae* Strain CSG20

**DOI:** 10.3390/s131115242

**Published:** 2013-11-07

**Authors:** Yun Fong Ngeow, Huey Jia Cheng, Jian Woon Chen, Wai-Fong Yin, Kok-Gan Chan

**Affiliations:** 1 Department of Medical Microbiology, Faculty of Medicine, University of Malaya, Kuala Lumpur 50603, Malaysia; E-Mail: yunngeow@um.edu.my; 2 Division of Genetics and Molecular Biology, Institute of Biological Sciences, Faculty of Science, University of Malaya, Kuala Lumpur 50603, Malaysia; E-Mails: chenghj90@gmail.com (H.J.C.); cjw246@hotmail.com (J.W.C.); yinwaifong@yahoo.com (W.-F.Y.)

**Keywords:** *Klebsiella pneumonia*, multidrug resistant, mass spectrometry, MALDI-TOF, *N*-hexanoylhomoserine lactone (C6-HSL), clinical pathogen, quorum sensing

## Abstract

*Klebsiella pneumoniae* is one of the most common Gram-negative bacterial pathogens in clinical practice. It is associated with a wide range of disorders, ranging from superficial skin and soft tissue infections to potentially fatal sepsis in the lungs and blood stream. Quorum sensing, or bacterial cell-cell communication, refers to population density-dependent gene expression modulation. Quorum sensing in Proteobacteria relies on the production and sensing of signaling molecules which are mostly *N*-acylhomoserine lactones. Here, we report the identification of a multidrug resistant clinical isolate, *K. pneumoniae* strain CSG20, using matrix-assisted laser desorption ionization-time-of-flight (MALDI-TOF) mass spectrometry. We further confirmed quorum sensing activity in this strain with the use of high resolution tandem liquid chromatography quadrupole mass spectrometry and provided evidence *K. pneumoniae* strain CSG20 produced *N*-hexanoyl-homoserine lactone (C6-HSL). To the best of our knowledge, this is the first report on the production of *N*-hexanoylhomoserine lactone (C6-HSL) in clinical isolate *K. pneumoniae*.

## 1. Introduction

*Klebsiella pneumoniae* is found ubiquitously in the natural environment and mammalian mucous membranes [1]. In humans, it forms part of the normal flora in the alimentary tract [2]. It is common hospital-acquired pathogen causing a variety of disorders, ranging from superficial wound infections to severe, life-threatening sepsis [3,4]. The most serious community-acquired infection is a fulminant pneumonia that is more common among chronic alcoholics, diabetics and older age groups with debilitating illnesses [5–7]. Risk factors for nosocomial infection or colonization include multiple antibiotic therapies, treatment in an intensive care unit and contamination of indwelling medical devices such as ventilator tubings, central venous and urinary catheters [8]. In the past few decades, extensive use of broad-spectrum antibiotics has led to the emergence of strains that show plasmid- or transposon-mediated resistance to multiple antibiotics including the β-lactam antibiotics, fluoroquinolones, aminoglycosides, cotrimoxazole and tetracyclines [9–12].

Quorum sensing (QS) typically refers to a process of bacterial cell-to-cell communication that relies on the production and detection of extracellular signaling molecules called autoinducers. In Gram-negative bacteria, autoinducers are mostly derivatives of an *N*-acylhomoserine lactone (AHL) backbone with species-specific substitutions [13]. QS allows bacterial cells to control gene expression collectively, and thus synchronize bacterial group behavior at a population density-dependent manner. QS-controlled phenotypic processes are unproductive unless a high density of bacterial cells acts in unison. The common goal of colonizing or infecting a host is a task that is often impossible for a single cell to achieve; a collective effort *via* the QS mechanism, however, will greatly enhance the chance of success. Many of the biological functions controlled by QS are associated with bacterial virulence, such as the production of extracellular polysaccharides [14], biofilm formation [15–17], iron binding and innate bactericidal activities [18]. On this basis, research in QS in clinical pathogens will allow a better understanding of virulence determinants production and the molecular basis of pathogenicity.

In this study, we examined the QS activity of a clinical isolate, *K. pneumoniae* strain CSG20, using a biosensor assay and high resolution tandem liquid chromatography quadrupole mass spectrometry for the detection and characterization of AHLs.

## 2. Experimental Section

### 2.1. Bacterial Strains and Culture Conditions

Strain CSG20 was isolated from the sacral wound swab of a patient who had undergone a craniotomy that led to a two-month post-operative recovery in the hospital. It was a non-motile, oxidase-negative and Gram-negative bacillus that grew as large, mucoid, lactose-fermenting colonies on Mac Conkey agar after 24 h incubation at 36 °C. Next, it was sub-cultured onto Tryptic Soy Agar (Scharlau, Barcelona, Spain) for bacterial identification. In the routine diagnostic laboratory, it was identified as *K. pneumoniae* by the IMVIC reactions (indole-negative, methyl red-positive, Voges Proskauer-negative and citrate-positive) and was found to be a producer of extended-spectrum β-lactamase (ESBL). The agar disk diffusion (Kirby-Bauer) test showed in vitro resistance to ampicillin, amoxicillin-clavulanate, ampicillin-sulbactam, cephalexin, cefuroxime and cotrimoxazole but susceptibility to sulperazone, imipenem, gentamicin, netilmicin and ciprofloxacin.

*Chromobacterium violaceum* CV026 was used as the QS biosensor. This is a double mini-T*n5* mutant derived from ATCC31532 that produces violacein pigment only in the presence of *N*–acyl side chains of 4–8 carbons [19]. It was grown in Luria Bertani (LB) broth (1% w/v tryptone, 1% w/v sodium chloride and 0.5% w/v yeast extract), or LB agar (LBA) (LB with addition of 1.5% w/v Bacto-agar) incubated at 28 °C.

### 2.2. Sample Preparation for MALDI-TOF MS and Data Analysis

A single colony of overnight culture strain CSG20 was smeared onto the MSP 96 target polished steel BC plate and subjected to MALDI-TOF MS analysis as reported previously [20]. It was analyzing using Microflex MALDI-TOF (Bruker Daltonik GmbH, Leipzig, Germany) bench-top mass spectrometer (equipped with UV laser at wavelength 337 nm) with the Bruker FlexControl software Version 3.3 (Build 108). The spectra were recorded in the linear positive ion mode and analyzed over a mass range of 2 to 20 kDa (acceleration voltage set at 20 kV). MALDI-TOF spot on the target plate was measured by the MBT-autoX.axe autoExecute method resulted from six series of 40 laser shots at different positions on the spotted product. The MALDI-TOF spectra were then analyzed in the Bruker MALDI Biotyper Real Time Classification (RTC) Version 3.1 (Build 65) software and presented with score-oriented dendrogram created by MALDI Biotyper MSP creation method (Bruker Daltonics, Bremen, Germany), where distance values are relative and routinely normalized to a maximum value of 1,000. Dendrograms were generated by similarity scoring of a set of mass spectra. The matching of unknown spectra to the main spectrum was evaluated based on dedicated score values and the results were reported as the best match to the Bruker database as per the manufacturer's manual [21].

### 2.3. Detection of AHL Production

AHL production in strain CSG20 was assayed by cross-streaking it perpendicular to *C. violaceum* CV026 on LB agar [22]. A purple pigmentation formed in *C. violaceum* CV026 suggests QS activity associated with the production of short-chain AHLs. E. carotovora *GS101 and* E. carotovora *PNP22 were used as positive and negative controls, respectively* [23].

### 2.4. AHL Extraction

AHL extraction from strain CSG20 was performed as reported previously [24,25]. In brief, the strain was grown overnight in LB medium (15 mL) buffered with 50 mM 3-[*N*–morpholino] propanesulfonic acid (MOPS) to pH 5.5 to prevent spontaneous degradation of AHLs [26]. The culture supernatant was extracted twice with acidified ethyl acetate (15 mL, 0.1% v/v glacial acetic acid) and evaporated to dryness under vacuum, resuspended in a minimal volume of acetonitrile and used for analysis in the high resolution triple quadrupole liquid chromatography (LC) tandem mass spectrometer (MS) to provide unequivocal confirmation of the presence of AHLs in strain CSG20. To identify AHLs via LC-MS/MS, we used synthetic AHLs, *N*-hexanoyl-L-homoserine lactone (C6-HSL) (obtained from Cayman Chemical, Ann Arbor, MI, USA) as standards and their spectra were compared to those of putative AHLs identified in strain CSG20. Stock solutions for standards (1 mg/mL) were prepared in acetonitrile and stored at −20 °C.

### 2.5. Triple Quadrupole Liquid Chromatography Mass Spectrometry (LC-MS/MS) Analysis

LC was carried out on the Agilent 1290 Infinity LC system (Agilent Technologies Inc., Santa Clara, CA, USA) coupled with Agilent ZORBAX Rapid Resolution High Definition SB-C18 Threaded Column (2.1 mm × 50 mm, 1.8 μm particle size). The flow rate was set at 0.3 mL/min (37 °C) and the injection volume was fixed at 2 μL. Mobile phases A and B referred to 0.1% v/v formic acid in water and 0.1% v/v formic acid in acetonitrile, respectively. The gradient profile used was as follows (time: mobile phase A: mobile phase B): 0 min: 80:20, 7 min: 50:50, 12 min: 20:80, and 14 min: 80:20. MS detection from UHPLC separated compounds was performed on the Agilent 6490 Triple Quadrupole LC/MS system (Agilent Technologies Inc., Santa Clara, CA, USA). Precursor ion-scanning experiments were performed in positive ion mode with Q3 set to monitor for *m*/*z* 102 and Q1 set to scan a mass range of *m*/*z* 80 to *m*/*z* 400. Molecular mass of *m*/*z* 102 refers to lactone ring thus indicating the presence of AHL. The LC/MS parameters were as follows: probe capillary voltage set at 3 kV, sheath gas at 11 mL/h, nebulizer pressure 20 psi, desolvation temperature at 200 °C. The Agilent MassHunter software was used for the MS data analysis. Analysis was based on retention index and the comparison of EI mass spectra with standards.

## 3. Results and Discussion

**Figure 1.**
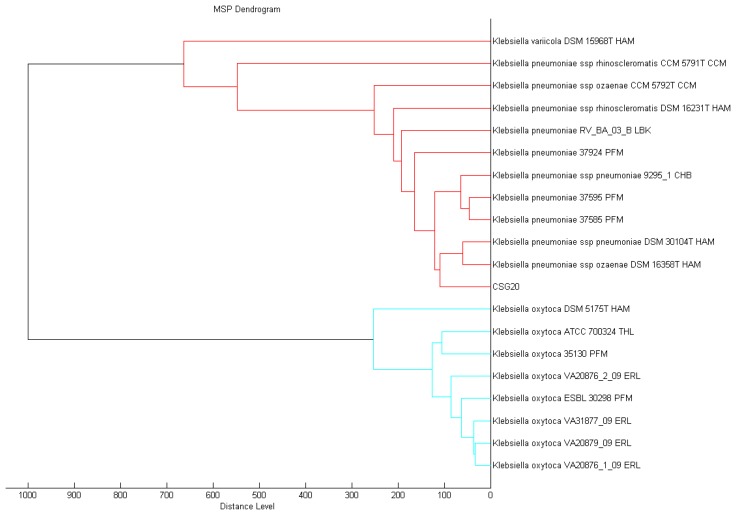
Score-oriented dendrogram of clinical isolate *K. pneumoniae* strain CSG2.

Conventional methods for the identification and typing of bacteria are often tedious and time-consuming and frequently give inconclusive results for rare and atypical species. In contrast, proteomic characterization with the use of mass spectrometry can provide fast and reliable identification of microorganisms including multidrug resistant clinical pathogens [27,28]. Proteomic phenotypes from MALDI-TOF MS have been employed effectively for microbial biotyping and the BioTyperTM MALDI-TOF MS fingerprinting system allows researchers to identify a wide spectrum of bacteria, yeasts and fungi [29]. With the spectra generated from our strain CSG20, and processed by standard pattern matching with MALDI-TOF Biotyper software, we were able to identify it with confidence as *K. pneumoniae* (Figure 1). We believe, as others have forecasted, that this technology will soon be the method of choice for pathogen identification in the diagnostic laboratories.

**Figure 2.**
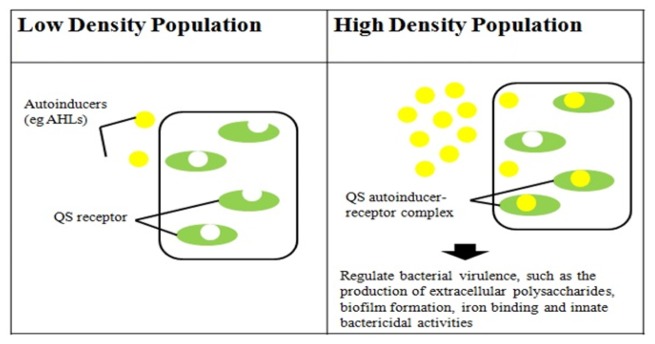
Diagram of quorum sensing.

**Figure 3.**
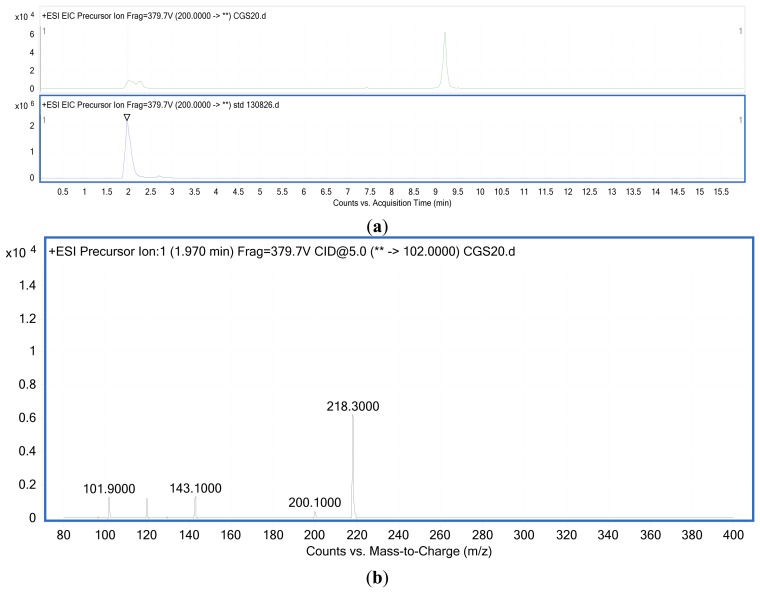
Mass spectra of AHLs extracted from *K. pneumoniae* strain CSG20 supernatant. (**a**) Total ion chromatogram of AHLs extract strain CSG20 and standard C6-HSL. (**b**) MS analysis of AHLs extract strain CGS20 shown product ion *m*/*z* 101.90 and precursor ion *m*/*z* 200.1 (Retention time: 1.970 min; Abundance: 439.96; Abundance %: 7.07).

Two major QS systems have been described in gram-negative bacteria, a type 1 system operating in intra-species communication and a type 2 system observed in inter-species communication. Type 1 QS is mediated by autoinducers known as AI-1 which are species-specific AHL's produced and regulated by AHL synthases encoded by the *luxI* gene [30,31]. The second protein in this system is the LuxR protein that detects AHL's and causes downstream change in gene expression [32] (Figure 2). Type 2 QS responds to a family of autoinducers known as AI-2 [33,34]. The AI-2 molecule is a furanosyl borate diester (Cao) produced by the LuxS protein encoded by the *luxS* gene [13,35]. Balestrino *et al.* [36] have reported the secretion of type 2 signaling molecules regulated by a homologue of the *luxS* gene, and the presence of AHL-lactonase, a quorum-quenching enzyme in *K. pneumoniae* [37–41]. De Araujo *et al.* [16] described the involvement of AI-2 autoinducers of *K. pneumoniae* in the regulation of biofilm formation. *K. pneumonia* strains producing AHLs have also been reported. Yin *et al.* [42] isolated them from the posterior dorsal surface of the tongue and demonstrated the production of *N*-octanoylhomoserine lactone (C8-HSL) and *N*-3-dodecanoylhomoserine lactone (C12-HSL) in these oral isolates. Our *K. pneumoniae* strain CSG20 produced C6-HSL (Figure 3).

It is possible that strains from different sites of infection show different AHL production profiles, just as different growth conditions in the laboratory can affect the type and amount of AHLs produced in bacteria [43]. It has been demonstrated, for instance, that *K. pneumoniae* and *K. oxytoca* strains produced AHLs only when grown in LB medium in microaerophilic but not under aerobic conditions [43]. However, we were able to detect AHLs in our strain CSG20 that was grown aerobically. This could be due to our use of a pH buffer, MOPS, in the LB growth medium. By acidifying the growth medium, MOPS can prevent the inactivation of AHLs by lactonolysis that occurs in alkaline conditions [26]. It is possible that MOPS also stabilized the AHLs produced in our CSG20 strain.

The natural phenomenon of QS has been the focus of recent research towards the discovery of novel, non-antibiotic-dependent approaches to anti-infective therapy [44–50]. The description of new AHL signals in a multidrug resistant *K. pneumoniae* strain in this paper will provide additional molecular targets towards the quest for QS-based alternative treatment.

## 4. Conclusions

Here, we were presented clinical isolate, *K. pneumoniae* strain CSG20 was produced C6-HSL. This funding will provide better understanding of QS activity of *K. pneumoniae* and may enable the anti-QS as one of the alternative anti-infective therapy.
